# Quantitative Control of Oxygen Non‐Stoichiometry and Negative Raman Chemical Shift During Topotactic Phase Transition in a Ca‐Doped BiFeO_3_ Thin‐Film

**DOI:** 10.1002/advs.202502439

**Published:** 2025-08-11

**Authors:** Heung‐Sik Park, Semin Cheon, Yong‐Jun Kwon, Minho Kang, Jeonghun Suh, Sang‐Youn Park, Yongsoo Yang, Chan‐Ho Yang

**Affiliations:** ^1^ Department of Physics Korea Advanced Institute of Science and Technology Daejeon 34141 Republic of Korea; ^2^ Center for Lattice Defectronics Korea Advanced Institute of Science and Technology Daejeon 34141 Republic of Korea; ^3^ Pohang Accelerator Laboratory Pohang University of Science and Technology Pohang 37673 Republic of Korea; ^4^ Graduate School of Semiconductor Technology School of Electrical Engineering Korea Advanced Institute of Science and Technology Daejeon 34141 Republic of Korea

**Keywords:** BiFeO3^*^, electrochemical titration, oxygen vacancy, raman spectroscopy, topotactic phase transition

## Abstract

Controlling the non‐stoichiometry is an effective way to tune physicochemical properties of functional oxides and explore novel physical phenomena in complex oxides. Therefore, quantitative control of oxygen non‐stoichiometry in perovskite oxides plays an important role in understanding the mechanism of topotactic phase transition and improving the applicability of electrochemical devices. Here, an electrochemical titration cell is fabricated to control the oxygen non‐stoichiometry of a Bi_0.4_Ca_0.6_FeO_3‐_ *_δ_* thin film grown on yttria‐stabilized zirconia substrate. The amount of oxygen pumped into the thin film depending on the applied voltage is quantitatively determined through chronoamperometry measurements. Non‐monotonic changes in lattice parameters are observed using X‐ray diffraction. Considerable negative Raman chemical shifts have occurred even in the range of small applied voltages that result in little changes in the non‐stoichiometry or average lattice parameters. These observations on chemo‐mechanical coupling can be interpreted as characteristics of vacancy‐ordered perovskite oxides. This work offers useful insights into the mechanism of topotactic phase transition in perovskite oxides.

## Graphical abstract

In an electrochemical titration cell utilizing a Ca‐doped BiFeO_3_ film, the oxygen stoichiometry was quantitatively controlled via electrical oxygen pumping. The structural and chemical changes were monitored through Raman spectroscopy and X‐ray diffraction. Notably, the observation of a negative Raman chemical shift upon oxidation suggests that this phenomenon may be a distinctive signature of systems featuring ordered oxygen vacancies.

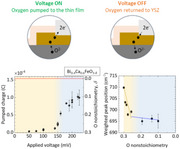

## 1 Introduction

Perovskite oxide thin films have attracted attention due to their highly tunable physical properties, such as mixed ionic‐electronic conduction,^[^ ^1^, ^2^ ^]^ magnetism,^[^ ^3^, ^4^ ^]^ and superconductivity.^[^ ^5^ ^]^ In many cases, oxygen vacancies as doubly charged donors affect those properties. Thus, oxygen non‐stoichiometry is an important parameter that can dramatically control the emerging properties of perovskite oxides. For example, superconductivity is observed in an infinite‐layer nickelate Nd_0.8_Sr_0.2_NiO_2_, which is synthesized by topotactic reduction on its perovskite precursor phase.^[^ ^5^ ^]^ Various functionalities associated with resistive switching^[^ ^6^, ^7^ ^]^ or electrochromism^[^ ^8^, ^9^ ^]^ originate from the reversible change of their corresponding properties, such as electrical resistance or optical absorption by control of oxygen non‐stoichiometry. Controlling the amount of a specific type of substance such as oxygen and hydrogen induces a structural change while maintaining its overall structural framework, which is called topotactic phase transition. A popular type of topotactic transition is the reversible transition between perovskite and Brownmillerite structures (i.e., the transition between ABO_3_ and ABO_2.5_), as in SrFeO_3‐δ_.^[^ ^10^, ^11^ ^]^ The switchability leads to their potential for various technological applications, such as resistive random access memories,^[^ ^12^, ^13^ ^]^ electrochemical random access memories (ECRAM),^[^ ^14^, ^15^ ^]^ and electrochromic windows.^[^ ^16^, ^17^ ^]^ Thus, it is crucial to quantitatively control oxygen non‐stoichiometry in a reversible way.^[^ ^1^ ^]^

Various experimental techniques have been utilized to adjust the non‐stoichiometry of oxides. Ionic liquid gating is a popular way to trigger topotactic transitions via injection and extraction of hydrogen or oxygen.^[^ ^18^, ^19^, ^20^, ^21^ ^]^ Metal hydrides such as NaH or CaH_2_ are strong reagents for inducing oxygen reduction.^[^ ^22^, ^23^ ^]^ Some perovskite manganites or cobaltates can change their oxygen non‐stoichiometry by thermal annealing in elevated temperatures.^[^ ^3^, ^24^ ^]^

Electrochemical titration cells based on pure ionic conductors such as yttria‐stabilized zirconia (YSZ) enable reversible and quantitative control of the oxygen non‐stoichiometry in perovskite oxides. The oxygen non‐stoichiometry of SrCoO_3‐_ *_x_* and Sr(Ti,Fe)O_3‐_ *_x_* thin films grown on YSZ substrates can be manipulated by external electrical potentials.^[^ ^1^, ^25^ ^]^ The amount of exchanged oxygen ions can be quantitatively determined by chronoamperometry. YSZ‐based perovskite oxide thin‐film systems are not only a platform for fundamental research on electrochemical titration cells but also constitute a part of various memory or energy devices such as ECRAM^[^ ^26^ ^]^ and solid oxide fuel cells.^[^ ^27^, ^28^ ^]^ Thus the discovery using this system can be directly applicable to real device applications.

Topotactic phase transition processes accompany not only stoichiometric changes but also changes in other material properties such as lattice parameters through chemical strain.^[^ ^29^ ^]^ Understanding the chemo‐mechanical coupling is crucial for developing more durable electrochemical devices, since the volume changes during operations are detrimental to device stability.^[^ ^30^, ^31^, ^32^ ^]^ The introduction of oxygen vacancies typically expands the crystal lattice in oxides. Therefore, the topotactic transition processes have been usually tracked by in situ X‐ray diffraction. When oxygen‐deficient perovskite oxides with an oxygen vacancy order, such as Brownmillerite, are oxidized during a topotactic phase transition, an abrupt decrease in lattice parameters is observed at the moment the oxygen vacancy order melts. However, it is not convenient to probe the topotactic transition processes in a spatially resolved manner due to the difficulty of focusing the X‐ray beam below the scale of a few micrometers.^[^ ^33^ ^]^

Recently, Raman microscopy has attracted attention as a fast, localized, and non‐destructive tool for measuring the chemomechanical coupling of perovskite oxides.^[^ ^23^, ^34^, ^35^, ^36^ ^]^ Sediva et al. correlate Raman vibrational characteristics with the oxygen non‐stoichiometry of Sr(Ti,Fe)O_3‐_ *_x_* where oxygen vacancies are randomly located without oxygen vacancy ordering.^[^ ^25^ ^]^ The interrelation between lattice vibrational frequency and oxygen non‐stoichiometry can be expressed as the Raman chemical shift constant defined as:

(1) $$\gamma_{C}=-\frac{1}{\omega_{0}\beta_{C}}{\left(\frac{\partial \omega }{\partial \delta }\right)}_{T,P}$$

where *ω* is the vibrational frequency at standard conditions, *δ* is the oxygen non‐stoichiometry, and β_*C*_ is the chemical expansivity defined as:

(2) $$\beta_{C}=\frac{1}{V}{\left(\frac{\partial V}{\partial \delta }\right)}_{T,P}$$

Bi_1‐_ *_x_* Ca*_x_* FeO_3‐_ *_δ_* is a mixed ionic‐electronic conductor having fast oxygen ionic diffusivities at relatively low temperatures (< 400 °C).^[^ ^37^ ^]^ The low‐temperature ionic diffusion is related with their vacancy ordered structure. Oxygen vacancies in Bi_1‐_ *_x_* Ca*_x_* FeO_3‐_ *_δ_* are accumulated in the planes that are periodically repeated along certain directions.^[^ ^38^ ^]^ The activation energies for oxygen ionic diffusivity of Bi_1‐_ *_x_* Ca*_x_* FeO_3‐_ *_δ_* grown on SrTiO_3_ (001) substrates are the lowest near *x* = 0.45 that is a compositional phase boundary between two phases having different directions for oxygen vacancy channels.^[^ ^39^ ^]^ Since aliovalent doping of Ca^2+^ cations generates oxygen vacancies, whose amount is proportional to Ca doping ratio (*x*), i.e., *δ* = *x*/2, while maintaining the valence state of Fe^3+^. The spontaneous oxygen vacancies produced by aliovalent doping are thermodynamically stable. Thermal annealing at 800 °C under a highly oxidizing atmosphere (125 atm) cannot eliminate its oxygen non‐stoichiometry.^[^ ^40^ ^]^ On the other hand, their oxygen non‐stoichiometry can be readily changed by the application of an electrical potential.^[^ ^37^, ^39^ ^]^

In this work, we quantitatively control oxygen non‐stoichiometry of a Bi_0.4_Ca_0.6_FeO_3‐_ *_δ_* (BCFO) perovskite oxide thin‐film grown on a YSZ substrate. Topotactic phase transition processes of the thin‐film are tracked by XRD and Raman microscopy.

## 2 Results and Discussion

**Figure 1:**
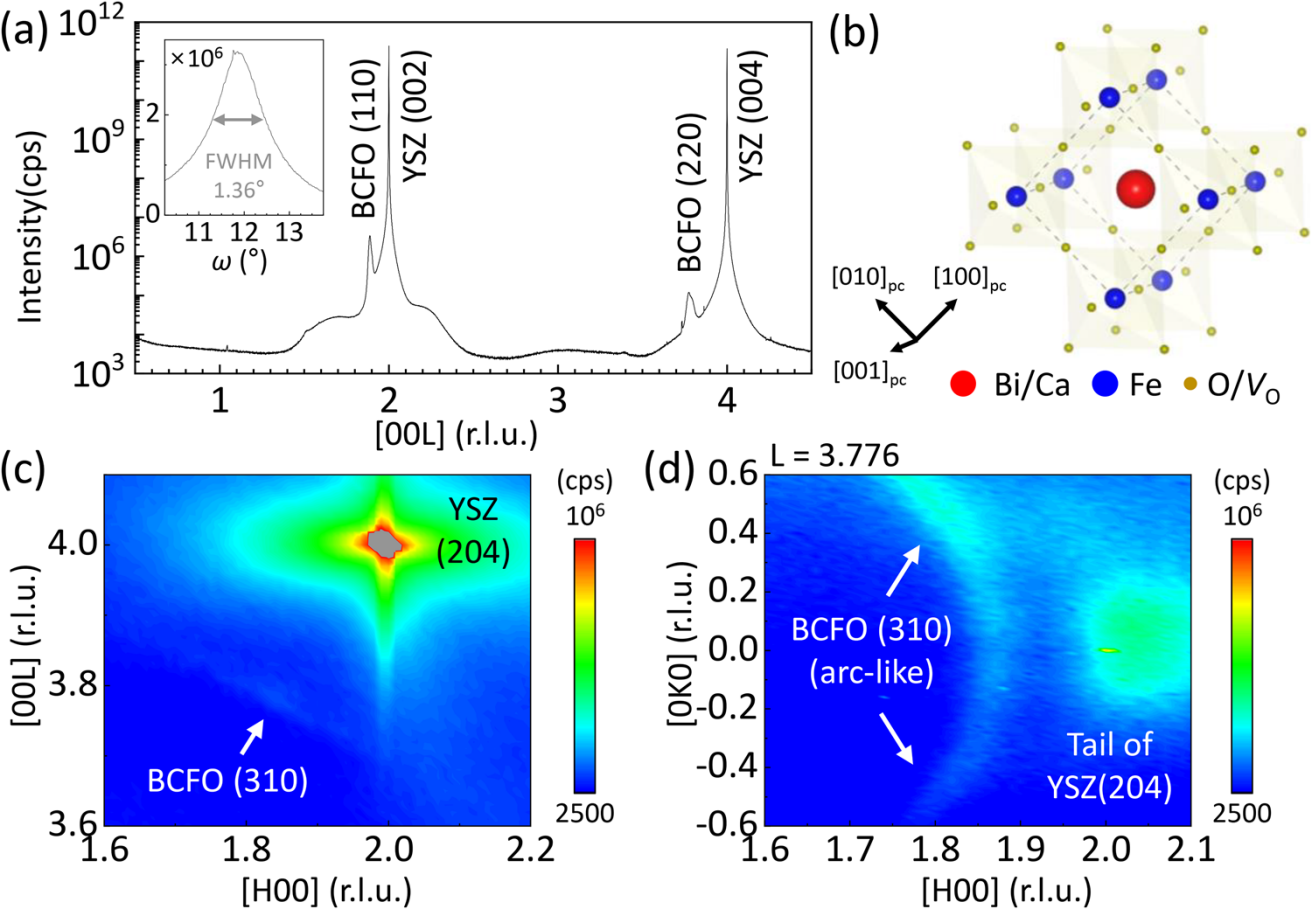
Structural charactersiation of a BCFO thin‐film grown on YSZ substrate. a) 00L scan for the BCFO/YSZ. Inset represents *ω* rocking curve at BCFO (110). b) Schematic representation of the crystal structure of BCFO (110). c) Reciprocal space mapping (HL scan) near BCFO (310). d) Reciprocal space mapping (HK scan) near BCFO(310).

**Figure 2:**
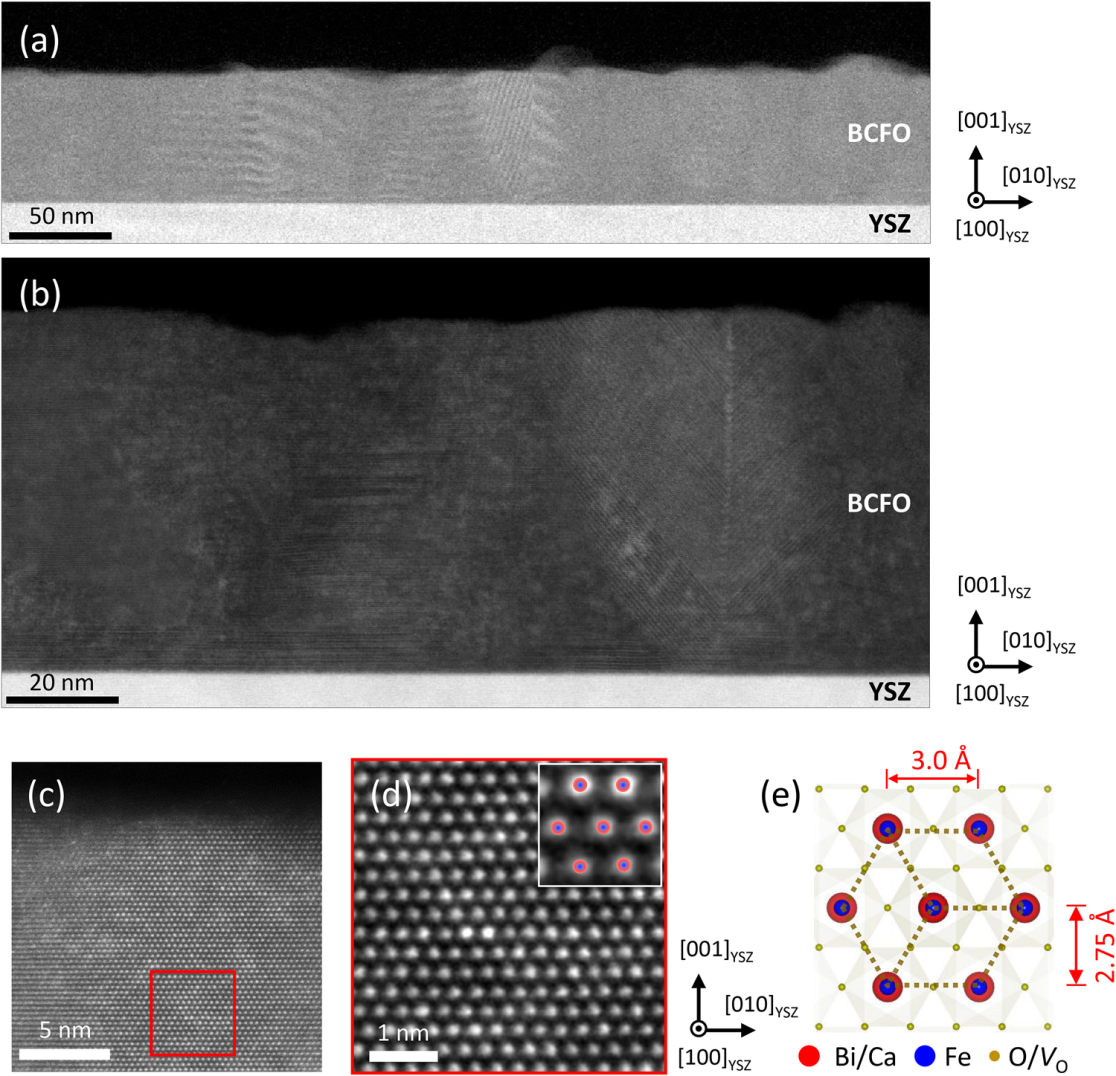
ADF‐STEM images of a BCFO thin‐film. a) A low‐magnification image showing the overall morphology of the BCFO film, which is composed of multiple grains (tens of nanometers in size) with varying in‐plane orientations. b) A medium‐magnification image of the BCFO film, showing more detailed structure within individual grains. c) An atomic resolution image near the surface of the film. d) A magnified view of the region within the red square in (c). e) A schematic representation of the atomic position of BCFO is shown in the inset of (d), consistent with [1‐11]_pc_ zone axis.

A BCFO thin‐film was grown on a YSZ (001) substrate by pulsed laser deposition (see the Experimental section for the details). As a result of the aliovalent doping the as‐grown BCFO thin‐film contains *δ* = 0.3 of the oxygen non‐stoichiometry that is half of the Ca‐doping ratio (i.e.*, x* = *δ*/2 = 0.6). XRD 2*θ*‐*ω* scan of the thin‐film indicates that the epitaxial BCFO film is grown along a pseudocubic [110] ([110]_pc_) direction (**Figure** **1**a), with a lattice spacing of ≈2.71 Å. It is believed that the broad and weak satellite peaks around the BCFO peaks arise from the presence of oxygen vacancy ordering planes that repeatedly emerge along the out‐of‐plane direction of the sample, exhibiting a periodicity that lacks full coherence. Figure 1b represents schematic illustration about crystal structure of perovskite oxide (110) grown on YSZ (002) substrate.^[^ ^41^ ^]^ The perovskite‐structured film is not fully strained to the fluorite‐structured substrate (Figure 1c). Moreover, the [001]_pc_ direction of the BCFO (110) domains is not consistently aligned parallel to the [100] direction of the YSZ substrate ([100]_YSZ_) (Figure 1d). Instead, the domains exhibit various degrees of in‐plane orientation, characterized by the azimuthal ϕ, which is defined as the angle between the [001]_pc_ direction of BCFO (110) and the [100]_YSZ_ directions (Figure S1a,b, Supporting Information). Figure S1a (Supporting Information) shows several possible zone axes schematics for different values of ϕ. To further examine the atomic structure of the BCFO film, cross‐sectional transmission electron microscopy (TEM) imaging was conducted. **Figure** **2**a,b presents low‐magnification images acquired using annular dark‐field scanning TEM (ADF‐STEM). The film is neither epitaxial nor single‐crystalline, due to the substantial lattice mismatch between the perovskite‐structured thin‐film and the fluorite‐structured substrate. However, extensive TEM measurements across various regions of the film consistently reveal an out‐of‐plane spacing of ≈2.75 Å across different domains (Figures S1c and S2a–l, Supporting Information). For example, atomic‐resolution images shown in Figure 2c–e reveal a well‐defined hexagonal lattice with an out‐of‐plane spacing of ≈2.75 Å. Given that this value closely matches the *c*‐axis lattice parameter of BCFO (110), it is reasonable to attribute this observation to the Grenier‐like phase viewed along the [1‐11]_pc_ zone axis of the film (ϕ = 54.7°), grown along the [110]_pc_ direction, rather than to assign it to another phase such as a hexagonal phase. These findings are further supported by the reciprocal space mapping results, which confirm that the BCFO (110) planes exhibit diverse in‐plane orientations within the film. Additional structural features suggest the presence of ordered lattice modulation along the film growth direction. Specifically, Figures S2e,g (Supporting Information) show alternating high‐ and low‐intensity atomic layers, indicative of a superlattice structure. Such contrast is characteristic of ordered oxygen vacancy arrangements, as observed in oxygen‐deficient perovskite phases like the Grenier phase.^[^ ^42^ ^]^ For a more detailed atomic‐level model of the topotactic transition in a BCFO thin film, please refer to Ref. [39]. While randomly distributed oxygen vacancies typically lead to uniform lattice expansion, periodic ordering of vacancies can give rise to superstructure reflections and distinct intensity modulations.^[^ ^38^ ^]^ It is important to note that cross‐sectional TEM imaging only reveals such ordering when the vacancy channels are aligned with the electron beam direction. Therefore, the observed contrast strongly suggests the presence of oxygen vacancy ordering in the pristine BCFO film.

**Figure 3:**
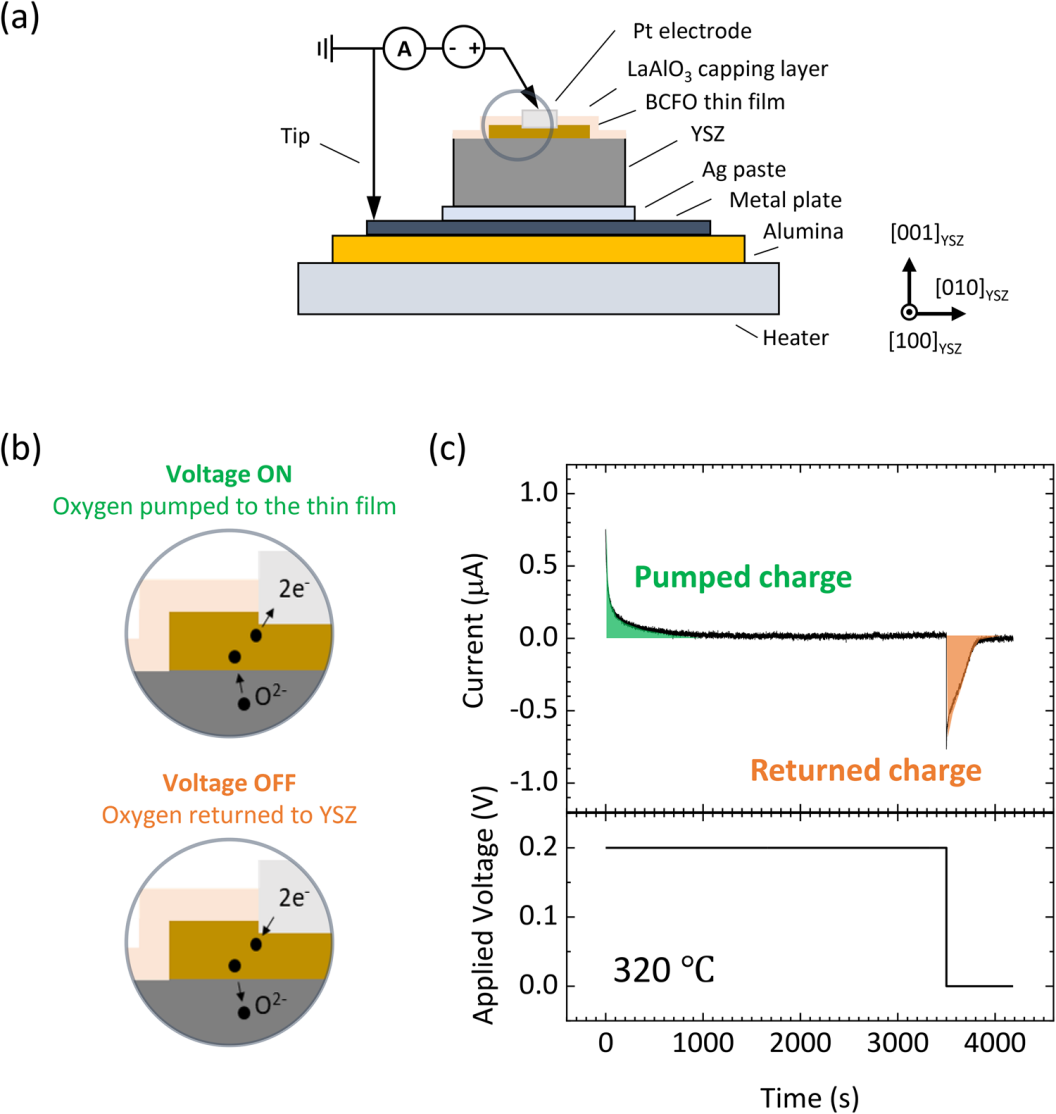
Electrochemical titration cell for oxygen pumping. a) Schematic illustration with a side view of the electrochemical titration cell. b) Schematic illustration of the oxygen pumping and returning depending on the voltage application. c) A chronoamperometry measurement result with monitoring oxygen pumping and returning. The colored areas represent the total pumped charge and the total returned charge after the applied voltage was changed.

A surface topographic image measured by atomic force microscopy is shown in Figure S3 (Supporting Information). A root‐mean‐square roughness of 1.62 nm is determined based on the image. **Figure** **3**a represents a schematic illustration of the electrochemical titration cell. The capping layer of LaAlO_3_ was deposited above the BCFO thin‐film to prevent oxygen exchange between the thin‐film and the atmosphere.^[^ ^43^ ^]^ Pt top electrode was deposited above the BCFO thin‐film after removing the capping layer. YSZ substrate was attached to a stainless steel metal plate with silver paste. When a positive voltage is applied to the top electrode while the metal plate acts as a ground electrode, oxygen is pumped to the thin‐film, since mobile oxygen ions within an oxide are negatively charged. When the voltage is turned off, oxygen diffusively returns back to YSZ (Figure 3b). Figure 3c shows a result of the chronoamperometry measurement acquired at an applying voltage of 0.2 V at 320 °C. The area under the current‐time curve represents the total electrical charge of oxygen pumped. Similarly, the area under the curve immediately after turning off the voltage corresponds to the total charge of oxygen returned.

**Figure 4:**
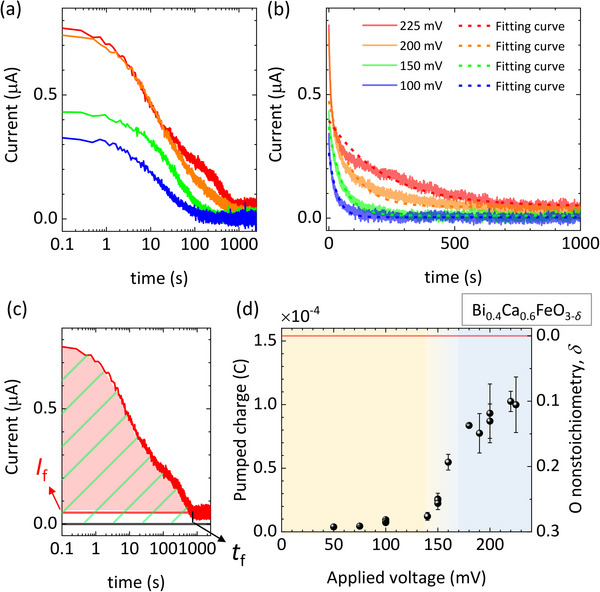
Chronoamperometry measurements and the determination of oxygen non‐stoichiometry. a,b) Real‐time currents during oxygen pumping with various applied voltages. Time is expressed in a logarithmic scale (a) or in a linear scale (b). The fitting curves are based on the equivalent circuit model shown in Figure S4c (Supporting Information). c) Determination of oxygen non‐stoichiometry from the chronoamperometry. The red area represents the minimum estimated pumped charge, and the green dashed area represents the maximum estimated pumped charge. d) Oxygen non‐stoichiometry obtained in a saturated state as a function of applied voltage.

**Figure** **4**a,b shows the chronoamperometry curves obtained at four different applied voltages, displayed in logarithmic and linear scales, respectively. For a given applied voltage, the current is initially high and then decreases over time. This decaying phenomena can be explained with the equivalent circuit shown in Figure S4a (Supporting Information) according to Baumann et al.^[^ ^44^ ^]^ Assuming that the oxygen stoichiometry variation at the interface between the film and the substrate is much smaller than the variation at the film for the simple treatment of the model, the equivalent circuit can be represented as Figure S4b (Supporting Information). Under constant applied voltage V, the time‐dependent currents follow exponential decay (Figure S4c, Supporting Information). By fitting the current‐time curves in Figure 4b to this model, we found that the fittings are reasonable for applied voltages below 150 mV. However, at 200 and 225 mV, the experimental data deviate significantly from the model, indicating that the equivalent circuit is no longer valid under these conditions. This discrepancy likely originates from a phase transition in the thin‐film during the pumping process, which causes substantial changes in one or more of the thin‐film‐related circuit elements‐namely, C_chem_, R_surface_, or R_interface_. After sufficient time from voltage application, the current level converges to a certain value that is nearly zero at low voltages (e.g., 100 mV) and to a non‐zero value at higher voltages (e.g., 225 mV). Although the transient electrical current in the initial stage is due to the pumping of oxygen anions, the current after the current converges to a certain value (*I*_f_) is thought to arise from oxygen exchange between the BCFO film and the atmosphere. In spite of the existence of a capping layer, the large amount of oxygen non‐stoichiometry change from the pristine state can lead to strong diffusion of oxygen from the thin‐film into the atmosphere. The positive *I*_f_ value causes an overestimation of the total charge of the pumped oxygen (Figure 4c). However, it is uncertain how large the *I*_f_ value is during the chronoamperometry measurement. An appropriate definition of the amount of the pumped charge and its error bar is required for quantitative and systematic measurements. Since it is reasonable to consider that the current associated with the atmospheric oxygen exchange becomes larger when more oxygen ions are pumped into the film as time goes on, the maximum amount of the atmospheric oxygen exchange would be *I*_f_ × *t*_f_, where *t*_f_ is the time when the current saturates to *I*_f_. So, the minimum amount of pumped charge is

(3) $$\int_{0}^{t_{f}}I\left(t\right)dt-I_{f}\times t_{f}$$

when *I*_f_ × *t*_f_ is the real current flowed due to the atmospheric oxygen exchange. On the other hand, the maximum amount of pumped charge is

(4) $$\int_{0}^{t_{f}}I\left(t\right)dt$$

when no current flowed due to the atmospheric oxygen exchange. Thus, we define the difference (*I*_f_ × *t*_f_) as an error bar for the determination of the amount of pumped charge. We define the amount of pumped charge as the average value of the maximum and the minimum:

(5) $$q_{p}=\int_{0}^{t_{f}}I\left(t\right)dt-\frac{I_{f}\times t_{f}}{2}$$

Figure 4d represents the pumped charge of oxygen (with the expression of the error bar as defined) as a function of an applied voltage. As the applied voltage increases, more oxygen ions are pumped into the BCFO thin‐film. The magnitudes of error bars, that are proportional to *I*_f_, tend to increase with stronger applied voltage. The pumped charge is converted to the change of oxygen non‐stoichiometry (Δδ) calculated as

(6) $$\Delta \delta =\frac{V_{\mathrm{unit}\;\mathrm{cell}}}{2eV_{\mathrm{film}}}q_{p}$$

where *V*_film_ is the volume of the BCFO thin‐film calculated from the geometric information of the device (Figure S5a,b, Supporting Information) as follows:

(7) $$\begin{array}{ccc} V_{\mathrm{film}} & = & \left(\mathrm{Volume}\;\mathrm{of}\;\mathrm{BCFO}\;\mathrm{thin}\;\mathrm{film}\;\mathrm{before}\;\mathrm{Ar}\;\mathrm{ion}\;\mathrm{milling}\right)-\\ & & \left(\mathrm{Volume}\;\mathrm{loss}\;\mathrm{due}\;\mathrm{to}\;\mathrm{Ar}\;\mathrm{ion}\;\mathrm{milling}\right)=\\ & & \left(1050\;\mu m\times 1900\;\mu m\times 50\;\mathrm{nm}\right)-\\ & & \left(430\;\mu m\times 1600\;\mu m\times 20\;\mathrm{nm}\right)=8.6\times 10^{-14}m^{3} \end{array}$$

and *V*_unit cell_ is the volume of the pseudocubic unit cell as follows:

(8) $$\begin{array}{ccc} V_{\mathrm{unit}\;\mathrm{cell}} & = & a_{\mathrm{BCFO}}\times b_{\mathrm{BCFO}}\times d_{\mathrm{BCFO}}\\ & & =3.58\;Å\times 5.51\;Å\times 2.71\;Å=53.5\;{Å}^{3} \end{array}$$

where *a*_BCFO_, *b*_BCFO_, and *d*_BCFO_ are defined in Figure S5c,d (Supporting Information). The thickness is estimated to be ≈50 nm according to the Scherrer formula. *b*_BCFO_ and *d*_BCFO_ are determined from the reciprocal space mapping (Figure 1d) and *a*_BCFO_ are determined from the TEM measurement (Figure 2d,e). Resultant oxygen non‐stoichiometry due to pumping is thus calculated as *δ* = 0.3 − Δ*δ*. Interestingly, the oxygen non‐stoichiometry is being saturated to zero as a large voltage is applied, reasonably indicating a large amount of oxygen vacancies are removed due to the pumping and it is hard for the BCFO thin‐film to accommodate oxygen excess.

Another interesting feature in Figure 4d is the presence of an anomaly near 150 mV. When a voltage less than 150 mV is applied, a small amount of oxygen is pumped into the film. A significant amount of pumping (i.e., *Δδ* > 0.05) occurs at voltages greater than 150 mV. This kind of nonlinearity is similar with the topotactic oxidation from SrCoO_2.5_ to SrCoO_3_.^[^ ^1^ ^]^ In this report, the rapid increase of *Δδ* occurs when the Brownmillerite to perovskite transition begins with the collapse of the oxygen vacancy order. We infer that the electrochemical potential applied to the ordered phase does not primarily contribute to the insertion of new oxygen ions into the lattice, but rather plays a role in disrupting the existing vacancy ordering, by minimizing the lattice spacing difference between oxygen and vacancy layers.^[^ ^45^ ^]^ Accordingly, when the oxygen nonstoichiometry exceeds a critical threshold and induces an order–disorder transition, the chemical capacitance—which characterizes the system's responsiveness of oxygen nonstoichiometry to variations in electrochemical potential—may exhibit a sudden change. The applied voltage of 150 mV at 320 °C corresponds to *p*O_2_^eff^ / *p*O_2_^atm^ = 1.3 × 10^5^, as calculated using the Nernst equation

(9) $$\frac{pO_{2}^{\mathrm{eff}}}{pO_{2}^{\mathrm{atm}}}=\exp\left(\frac{4eV}{k_{B}T}\right)\;$$

where *p*O_2_^eff^, *p*O_2_^atm^ is an effective oxygen partial pressure of the lattice and an atmospheric oxygen partial pressure (0.21 atm). The notably elevated pressure in this case underscores the difficulty of achieving full oxidation and stabilizing the perovskite phase in BCFO compounds. Supporting this, a bulk study reported that annealing BCFO at 800 °C under 125 atm of oxygen resulted in minimal changes in oxygen nonstoichiometry, with less than 10% of the oxygen vacancies being eliminated.^[^ ^40^ ^]^

**Figure 5:**
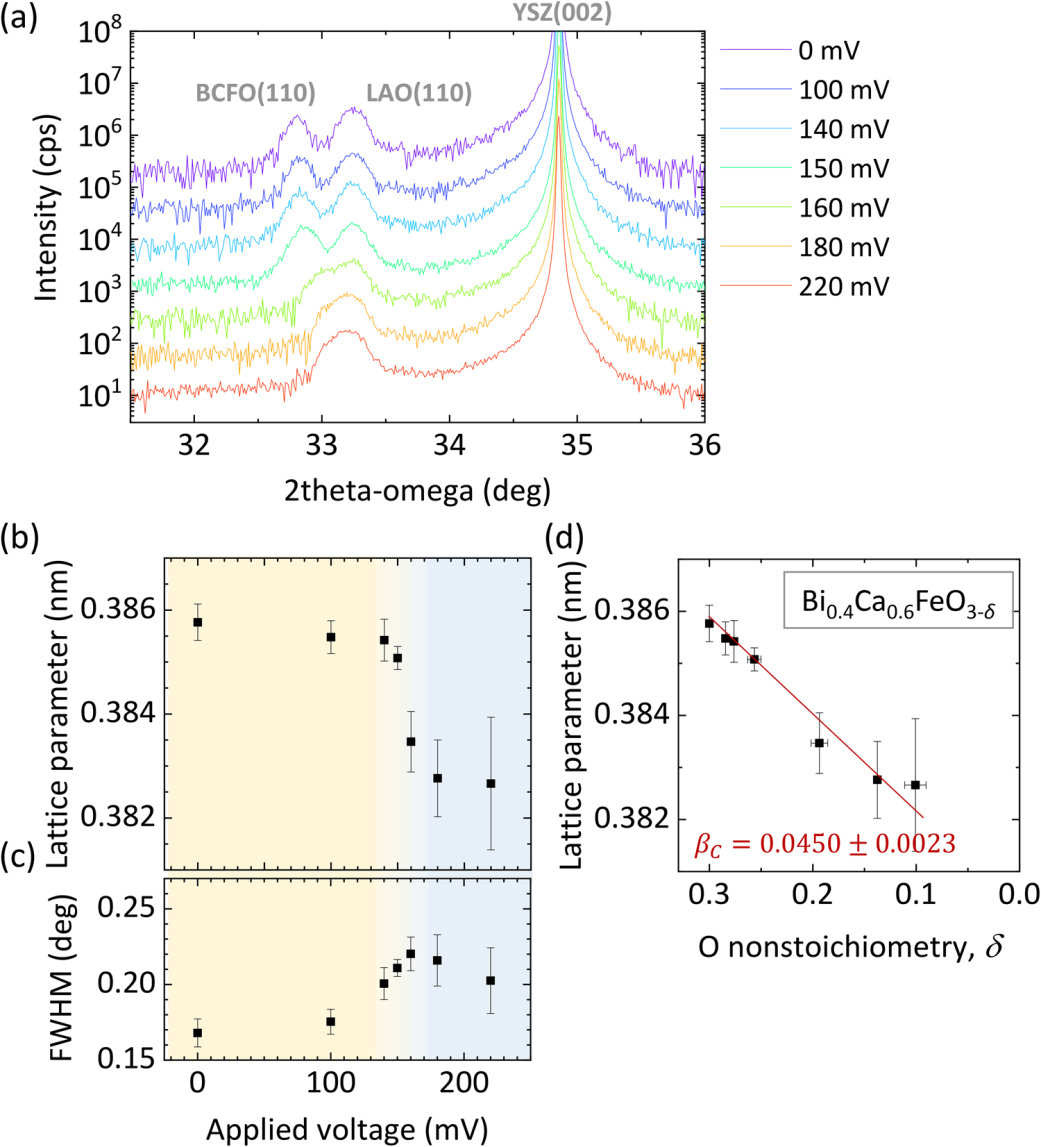
Monitoring of the chemical strains with XRD after the oxygen pumping. a) XRD 2θ‐ω scans at room‐temperature after the oxygen pumping. b) Pseudocubic lattice parameter of BCFO film after the oxygen pumping versus applied voltage. c) Change of the FWHM of the BCFO (110) peak. d) Correlation between the lattice parameter and oxygen non‐stoichiometry. The red line is guided by eye.

**Figure** **5**a shows XRD 2θ‐ω scans for samples after pumping with given applied voltages. XRD is measured at room‐temperature after quenching the samples while maintaining the applied voltages. Since the diffraction peak of the LaAlO_3_ capping layer overlaps with the peak of the BCFO thin‐film for large applied voltages, double Gaussian curve fitting was used to differentiate the peak positions. Figure 5b represents variation of the pseudocubic lattice parameter showing that the film contracts with the removal of oxygen vacancies. The pseudocubic lattice parameter is calculated as $\sqrt{2}$ times the lattice spacing determined from the 2*ϴ* value of the BCFO (110) peak. The contraction is not so dramatic in cases that the applied voltages are smaller than 150 mV. At the voltage larger than 150 mV, the lattice contraction becomes considerable being larger than the magnitude of fitting error bars. The full width at half maximum (FWHM) of the diffraction peak of the BCFO thin films show large values near the anomaly (i.e., near 150 mV) (Figure 5c). Figure 5d represents the correlation between the pseudocubic lattice parameter and oxygen non‐stoichiometry. Assuming that the volume change can be considered linearly proportional to the change in the pseudocubic lattice parameter, the chemical expansivity of the BCFO thin‐film is calculated to *β*_C_ = 0.0450 ± 0.0023.

**Figure 6:**
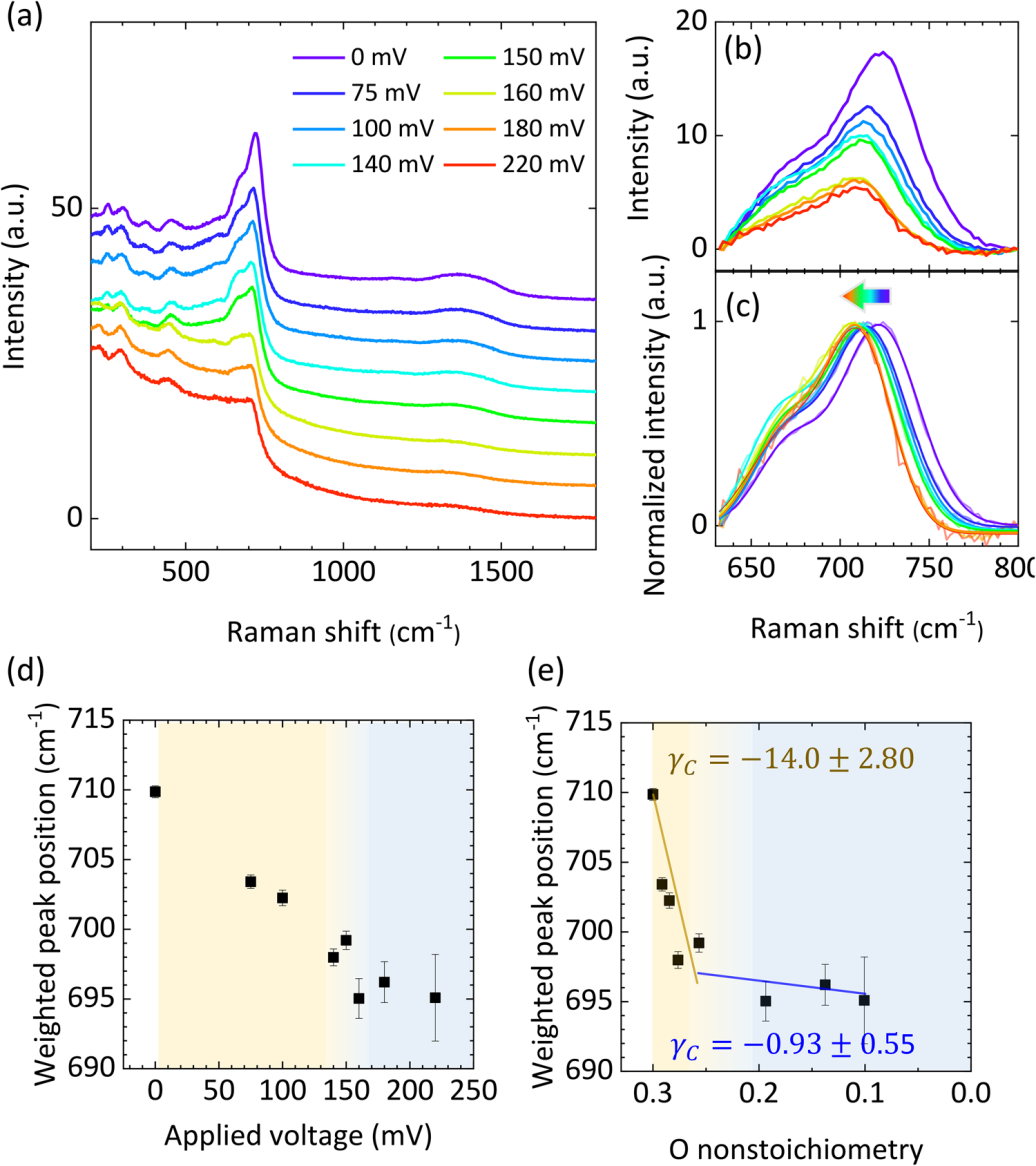
Monitoring of the Raman peak position after the oxygen pumping. a) Raman spectra of the BCFO thin‐film. Signals from the YSZ substrate are removed by post‐subtraction. b) Raman modes near 700 cm^−1^. Linear baseline is subtracted. c) Normalized Raman modes near 700 cm^−1^, showing the redshift of the peak position with the increase of applied voltage. d) Weighted peak position as a function of applied voltage. e) Weighted peak position as a function of oxygen non‐stoichiometry. Raman chemical shift constants are calculated at the early stage and the later stage. The brown and blue lines are guide lines.

Raman spectra are measured at room‐temperature for samples after quenching as with XRD measurements. **Figure** **6**a represents the Raman spectra of a BCFO thin‐film where the substrate signal is subtracted. We focus on the most prominent peaks near 700 cm^−1^ with a simple baseline subtraction using a straight line (Figure 6b). In many cases, the vibrational modes near 700 cm^−1^ of perovskite oxides correspond to in‐phase stretching modes of oxygen octahedra.^[^ ^25^, ^46^, ^47^ ^]^ The overall redshift of the Raman spectra due to oxidation with increasing applied voltage is clearly observed in the normalized spectra in Figure 6c. This indicates that the sign of the Raman chemical shift is negative unlike the report of the other perovskite oxide Sr(Ti,Fe)O*_x_*.^[^ ^25^ ^]^ Double Gaussian curves fit well with the subtracted spectra (Figure S6a, Supporting Information). Figure 4d shows weighted Raman peak position obtained from double Gaussian curve fitting as follows

(10) $$\frac{A_{1}}{A_{1}+A_{2}}x_{1}+\frac{A_{2}}{A_{1}+A_{2}}x_{2}$$

where *A*_1_, *A*_2_ are the integrated intensities of fitted Gaussian curves and *x*_1_, *x*_2_ are the peak positions of fitted curves. The values of *A*_1_, *A*_2_, *x*_1_, *x*_2_ are plotted in Figure S6b,c (Supporting Information). In contrast to the observations from chronoamperometry and XRD where pumping with applied voltages less than 150 mV induces no dramatic changes, significant redshift of the Raman peak position occurs even with small applied voltages. This result implies that the material undergoes a considerable change which is not accompanied with the changes in oxygen non‐stoichiometry and average *c*‐axis lattice parameter.

From the relationship between the applied voltage and the oxygen non‐stoichiometry in Figure 4d, the weighted peak position is plotted as a function of the oxygen non‐stoichiometry (Figure 6e). At the early stage of the oxidation process, corresponding to the pumping with applied voltages less than 150 mV, the redshift of the Raman peak position leads to a negative Raman chemical shift constant of *γ_C_* = ‐14.0 ± 2.80. The absolute value is much larger than the *γ_C_* = 2.05 ± 0.28 found in SrTi_0.7_Fe_0.3_O_3‐_ *_δ_*.^[^ ^25^ ^]^ On the other hand, the Raman chemical shift constant at the later stage of the oxidation process is calculated to *γ_C_* = ‐0.93 ± 0.55, which is close to zero. Given that the typical grain size is on the order of 50 nm, the grain boundary regions do not constitute a significant volume fraction that would critically influence our conclusions. Furthermore, no noticeable impurity phases were detected in the XRD or Raman spectra. Therefore, we believe that grain boundaries and minor impurity phases do not have a substantial impact on the analysis of chemomechanical coupling in this material.

The significant changes in Raman peak positions even at lower applied voltages may be attributed to the oxygen vacancy order‐disorder transition. In BCFO, where Ca‐doping ratio is 30%, grown on SrTiO_3_ substrate, negative Raman chemical shift during the oxygen vacancy order–disorder transition is observed by complementary measurements with TEM and Raman spectroscopy.^[^ ^45^ ^]^ Our chronoamperometry and XRD results show a similar tendency with the report from SrCoO*_x_* with the order–disorder transition between Brownmillerite and perovskite phases.^[^ ^1^ ^]^ In both experiments, the pumped charges and the lattice parameters are not changed dramatically at smaller voltages than 150 mV, by maintaining their ordered structure. When larger voltages than 150 mV are applied, the oxygen vacancy ordering is melted and oxygen vacancies act as homogeneously distributed point defects.

The possible origin of the negative Raman chemical shift may also originate from the vacancy‐ordered structure. In most of vacancy ordered perovskite‐type oxides, oxidation processes lead to a local expansion of the oxygen layer (e.g., octahedral layers in Brownmillerite oxides) while the average *c*‐axis lattice parameter gradually decreases.^[^ ^48^, ^49^ ^]^ It might give rise to the observed redshift since the increase of bond length typically leads to the decrease of Raman peak position in the viewpoint of the Raman chemical shift.^[^ ^25^ ^]^

## 3 Conclusion

In conclusion, we quantitatively controlled oxygen non‐stoichiometry of a perovskite BCFO thin‐film with a YSZ‐based electrochemical titration cell. The amount of pumped oxygen was determined by chronoamperometry as a function of applied voltage. Lattice contraction due to oxidation was also monitored by XRD. Both the chronoamperometry and XRD measurements indicate the existence of an anomaly near 150 mV, which may be related to an oxygen vacancy order–disorder transition. Even though there were little changes in oxygen non‐stoichiometry and lattice parameters at small applied voltages, the samples experienced significant changes in the Raman peak positions. This behavior may be interpreted as a feature of the oxygen vacancy order–disorder transition of perovskite‐type oxides. This work demonstrates deterministic control of oxygen non‐stoichiometry in perovskite‐type oxides and various ways to monitor material changes during the oxidation processes through chronoamperometry, XRD, and Raman spectroscopy.

## 4 Experimental Section

### Sample Preparation

Bi_0.4_Ca_0.6_FeO_3‐_ *_δ_* thin films were grown on YSZ (001) substrate by pulsed laser deposition using a KrF excimer laser (*λ* = 248 nm). The BCFO bulk target was prepared by a solid‐state reaction method. Bi_2_O_3_ (99.9%), CaO (99.95%), and Fe_2_O_3_ (99.9%) powders (Sigma–Aldrich) were mixed with 10% bismuth excess. The bulk pellet was calcined at ≈600 °C for 6 h. Then, the pellet was ground, and pressed into a button‐shaped target. The target was sintered at ∼650 °C for 6.5 h in air. The thin films were deposited at a growth temperature of 670 °C in an oxygen environment of 1 mTorr. The laser fluence and repetition rate were set to ≈1.5 J cm^−2^ and 10 Hz, respectively. The thin‐film was cooled to room‐temperature at a rate of 10 °C min^−1^ under an oxygen pressure of 450 Torr.

### X‐ray Diffraction and Reciprocal Space Mapping

X‐ray diffraction and reciprocal space mappings for the pristine sample were conducted at Beamline 3A of the Pohang Light Source II (PLS‐II). Monochromatic light with a wavelength of 1.126 Å was used for both (00L) scan and reciprocal space mappings. 2*θ*‐*ω* scans for samples after quenching were measured with an X‐ray diffractometer (PANalytical X'pert‐PRO MRD) with Cu K*_α_*_1_ radiation (*λ* = 1.5406 Å).

### Device Fabrication

The pumping device was patterned with UV‐lithography using AZ5214E photoresist (AZ Electronic Materials). The patterned sample was dry‐etched by argon‐ion millling. The LaAlO_3_ capping layer (≈30 nm thickness) was deposited on the whole sample surface over the substrate area as well as the remaining unetched BCFO regions by pulsed laser deposition. The growth temperature for the capping layer was 650 °C in an oxygen environment of 6 mTorr. The laser fluence and repetition rate were set to ≈1.5 J cm^−2^ and 2 Hz, respectively. The device was cooled to room‐temperature at a rate of 10 °C min^−1^ under an oxygen pressure of 450 Torr. Platinum was deposited for the top electrode by DC magnetron sputtering at an argon pressure of 5 mTorr at room‐temperature.

### Chronoamperometry Measurement

Constant voltage was applied with Keithley 230 programmable voltage source (Tektronix) to the platinum electrode through a gold‐coated probe tip on positioners (MS Tech). Another gold‐coated probe tip was contacted to the metal plate beneath the substrate acting as a ground electrode. The device was attached on a heating stage MHCS622V (Microptik) in air at 320 °C during the measurements. Current was monitored in real time, at 0.25 s intervals, with Keithley 2000 multimeter (Tektronix).

### Raman Spectroscopy

Raman spectra were measured at room‐temperature with alpha300 R Raman microscope (Oxford Instruments) for a device after quickly cooling from 320 °C at which the oxygen pumping process was made. The excitation laser wavelength was 532 nm and the power of the incident laser was adjusted to ≈1.0 mW. The polarization of incident light was aligned along the pseudocubic [100] axis (i.e.*, a*‐axis), while no analyzer was used. The scattered light was collected by the spectrometer UHTS 600 (Oxford Instruments) in the backscattering geometry. An 100× objective lens (Zeiss) with a numerical aperture of 0.9 was used.

### ADF‐STEM Measurements

A cross‐sectional lamella was prepared using a focused ion beam instrument (Helios G5 UX, Thermo Fisher Scientific). The thickness of the lamella was ≈35 nm. The specimen was subsequently transferred to a double‐tilt TEM holder for ADF‐STEM image acquisition using a double Cs‐corrected TEM (Spectra Ultra, Thermo Fisher Scientific). The microscope was operated at an accelerating voltage of 300 kV with a probe semi‐convergence angle of 21.4 mrad. The inner and outer angles of the ADF detector were set to 49 and 200 mrad, respectively. ADF‐STEM images of 2048 × 2048 pixels were acquired with a pixel dwell time of 4 µs and a screen current of 30 pA. The corresponding pixel sizes were 5.4 Å for Figure 2a, 0.78 Å for Figure 2b, 0.17 Å for Figure 2c, and 0.08 Å for Figure 2d. The positions of atomic columns in Figure S2e–h (Supporting Information) were traced using 2D Gaussian fitting. To calculate the intensity profiles along the atomic layers, the rotation angles between the atomic layers and the image axes were determined by least‐squares regression of the traced atomic positions. The intensity profiles were obtained by summing the image intensity along the direction parallel to the atomic layers.

## Conflict of Interest

The authors declare no conflict of interest.

## Author Contributions

H.‐S.P. conceived and designed the experiments. H.‐S.P. and J.S. synthesized the thin films. H.‐S.P. performed the chronoamperometry and Raman microscopy measurements. H.‐S.P., Y.‐J.K., M.K., and S.‐Y.P. carried out the XRD and reciprocal space mapping measurements. S.C. and Y.Y. conducted the TEM measurements. H.‐S.P. and C.‐H.Y. analyzed the data and wrote the manuscript. All authors discussed the results and contributed to the final version of the manuscript.

## Supporting information



Supporting Information

## Data Availability

The data that support the findings of this study are available from the corresponding author upon reasonable request.
