# Supplementary material for: Quantitative Control of Oxygen Non‐Stoichiometry and Negative Raman Chemical Shift During Topotactic Phase Transition in a Ca‐Doped BiFeO3 Thin‐Film
Source: Adv Sci (Weinh). 2025 Aug 11;12(40):e02439. doi: 10.1002/advs.202502439 (PMC12561177; doi:10.1002/advs.202502439)
Supplement: Supplementary file 1 — Supporting Information [file ADVS-12-e02439-s001.pdf]

Supporting Information

Quantitative control of oxygen non-stoichiometry and negative Raman chemical shift during topotactic phase transition in a Ca-doped $BiFeO_3$ thin film

Heung-Sik Park,[1,2] Semin Cheon,[1] Yong-Jun Kwon,[1,2] Minho Kang,[1,2] Jeonghun Suh,[1,2] Sang-Youn Park,[3] Yongsoo Yang,[1,4]* and Chan-Ho Yang[1,2,]*

[1]*Department of Physics, Korea Advanced Institute of Science and Technology, Daejeon 34141, Republic of Korea*

[2]*Center for Lattice Defectronics, Korea Advanced Institute of Science and Technology, Daejeon 34141, Republic of Korea*

[3]*Pohang Accelerator Laboratory, Pohang, 37673, Republic of Korea*

[4]*Graduate School of Semiconductor Technology, School of Electrical Engineering, Korea Advanced Institute of Science and Technology, Daejeon, 34141, Republic of Korea*

*Author to whom correspondence should be addressed: yongsoo.yang@kaist.ac.kr, chyang@kaist.ac.kr

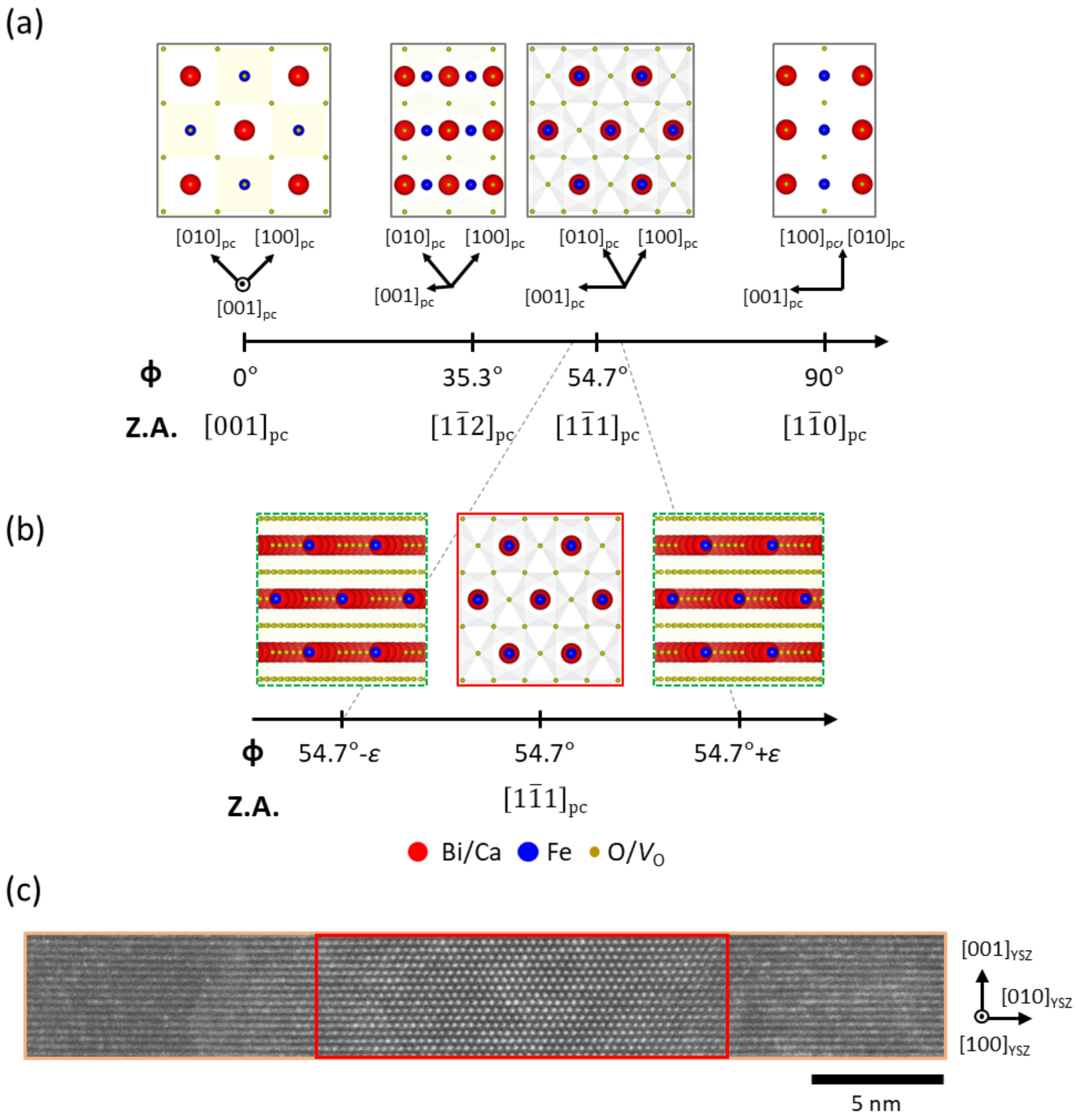

FIG. S1. BCFO (110) with various zone axes. (a) Schematic illustrations of BCFO (110) with special orientations that correspond to zone axes along $[001]_{pc}$, $[1\bar{1}2]_{pc}$, $[1\bar{1}1]_{pc}$, and $[1\bar{1}0]_{pc}$. ϕ denotes angle between $[001]_{pc}$ of BCFO (110) and zone axis ($[100]_{YSZ}$). (b) Schematic illustration of BCFO (110) with ϕ near 54.7°. (c) ADF-STEM image of BCFO (110), highlighting the on-zone region (within the red rectangle) and a slightly off-zone area near the $[1\bar{1}1]_{pc}$ zone axis.

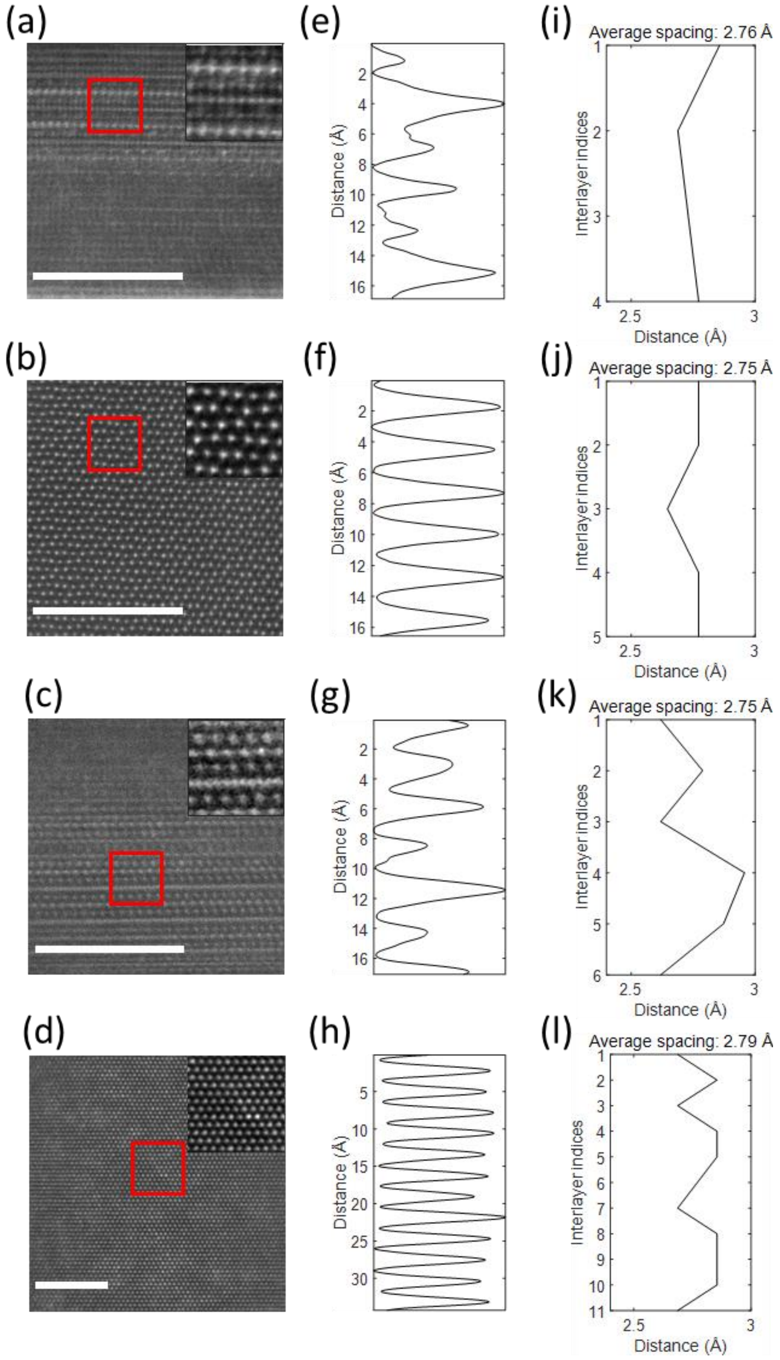

FIG. S2. (a-d) Atomic-resolution ADF-STEM images of a BCFO thin film acquired at different regions of the cross-section specimen. (a) near the substrate, (b) ~15 nm from the film-substrate interface, (c) ~25 nm from the interface, and (d) near the top surface. The total film thickness is approximately 50 nm. Insets show magnified views of the regions marked by red squares. (e-h) Intensity profiles obtained by summing the image intensity within each inset along the atomic layers. (i-l) Interlayer distances calculated from the corresponding intensity profiles in (e-h), with average values ranging from 2.75 to 2.79 Å. Scale bars: 5 nm.

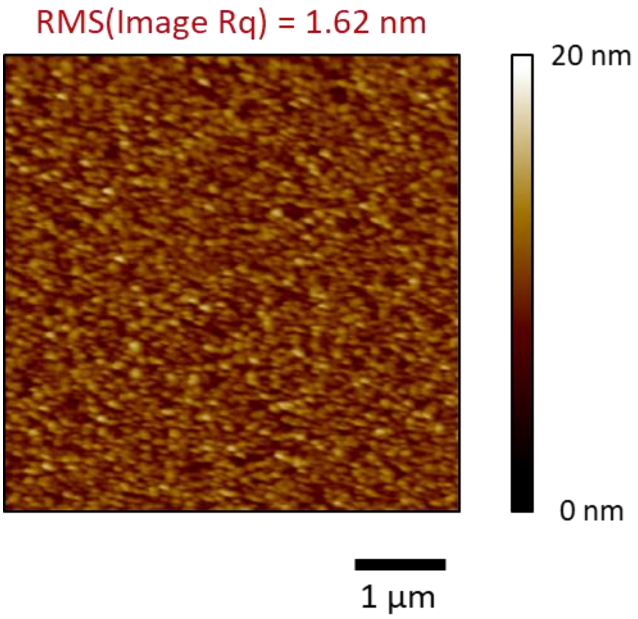

FIG. S3. Surface topographic image measured by atomic force microscopy of BCFO/YSZ thin film.

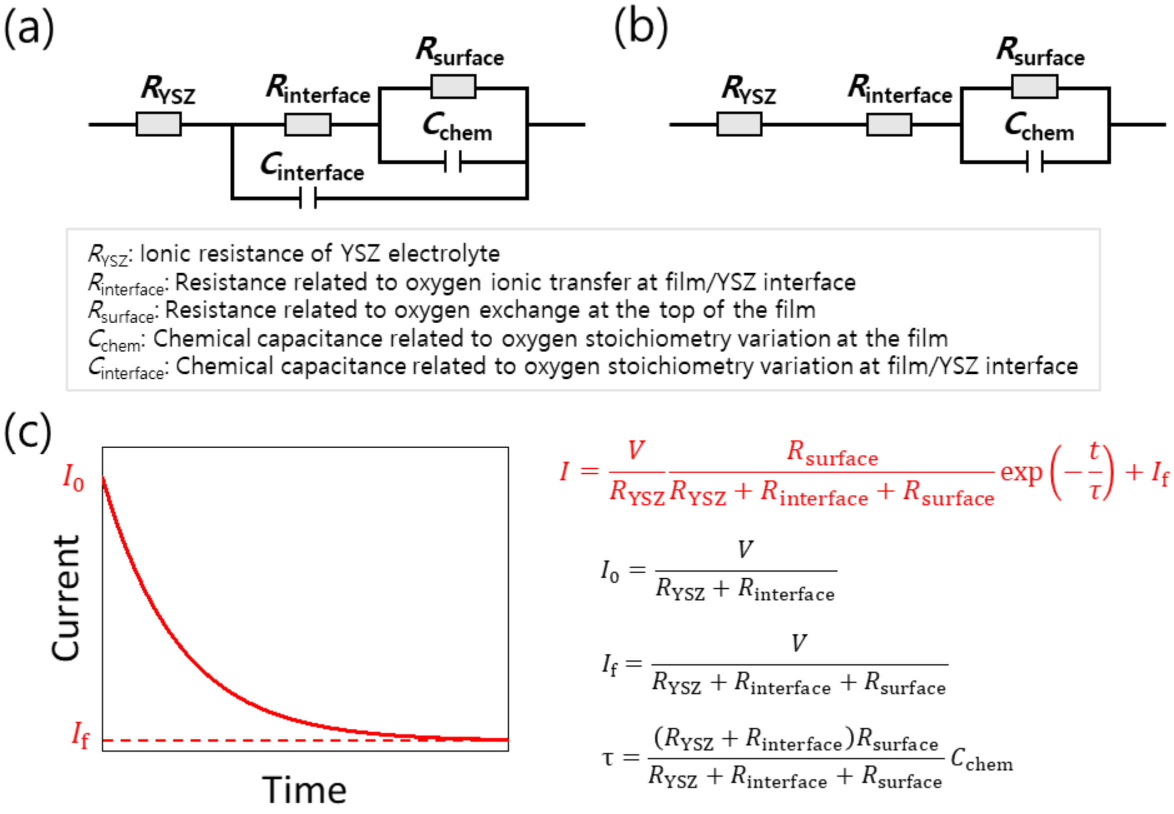

FIG. S4. Equivalent circuit model for a titration cell. (a) Equivalent circuit model for perovskite oxide grown on YSZ substrate. (b) Simpler equivalent circuit model with an assumption of negligible oxygen stoichiometry variation at a film/YSZ interface. (c) Current-time curves based on the model shown in (b).

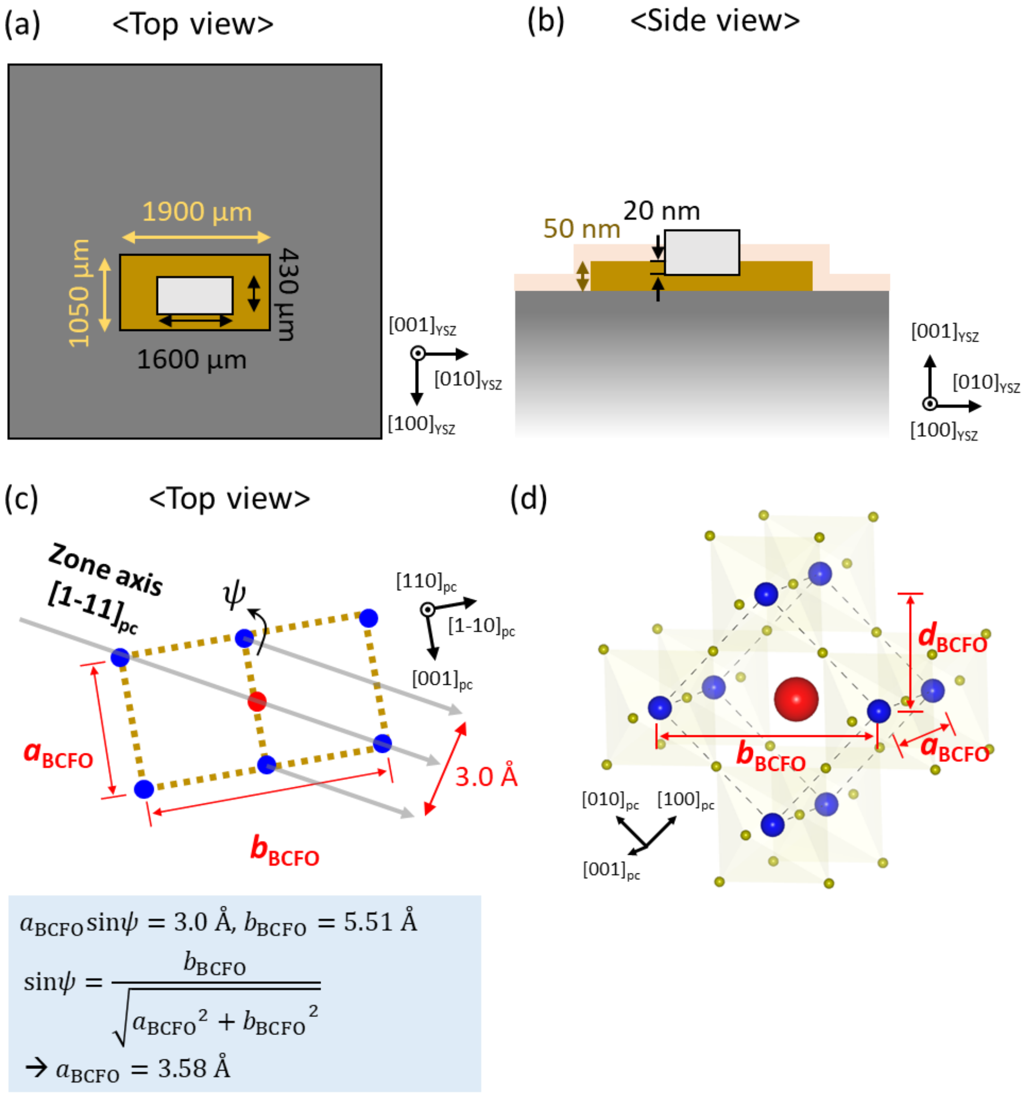

FIG. S5. Calculation of sample volume and unit cell volume. (a,b) Schematic illustrations representing the device geometry with a top view (a) and a side view (b). The BCFO film (brown) is covered by $LaAlO_3$ capping layer (pink) except for the Pt electrode region (pale grey). (c) Schematic illustrations of BCFO (110) and zone axis $[1\bar{1}1]_{pc}$ with a top view. (d) Schematic illustration of pseudocubic unit cell of BCFO and definitions of a_{BCFO}, b_{BCFO}, and d_{BCFO}.

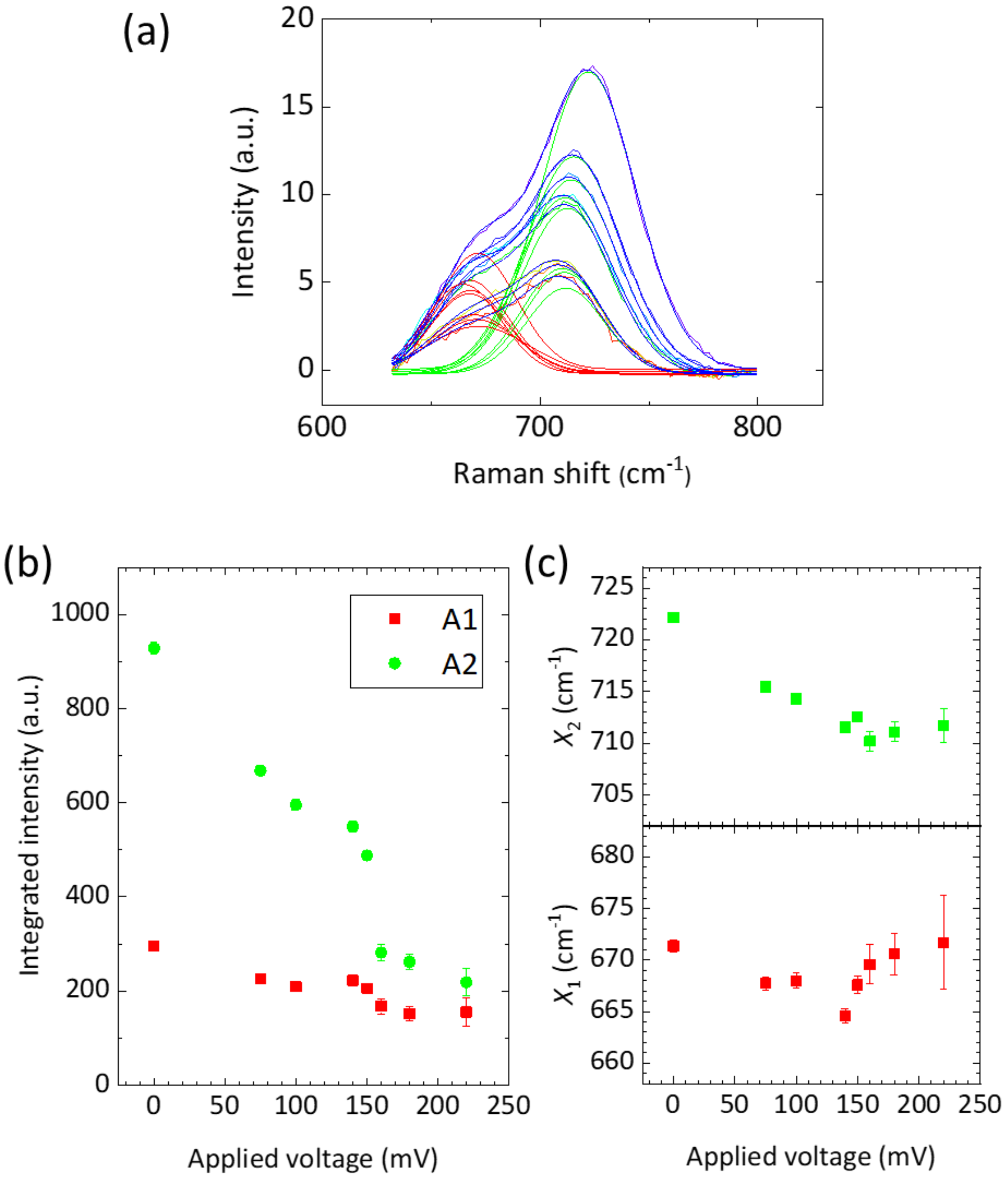

FIG. S6. (a) Fitting of Raman modes near 700 cm^{-1} with double Gaussian curves. (b) Integrated intensities of the two Gaussian curves used for fitting. (c) Peak centers of the Gaussian curves used for fitting.
